# Commercially Available Mobile Phone Headache Diary Apps: A Systematic Review

**DOI:** 10.2196/mhealth.3452

**Published:** 2014-08-19

**Authors:** Amos S Hundert, Anna Huguet, Patrick J McGrath, Jennifer N Stinson, Mike Wheaton

**Affiliations:** ^1^IWK Health CenterHalifax, NSCanada; ^2^Department of Community Health and EpidemiologyDalhousie UniversityHalifax, NSCanada; ^3^Departments of Pediatrics and PsychiatryDalhousie UniversityHalifax, NSCanada; ^4^Hospital for Sick ChildrenToronto, ONCanada; ^5^Lawrence S. Bloomberg Faculty of NursingUniversity of TorontoToronto, ONCanada

**Keywords:** headache, diary, apps, smartphone, mobile phone, technology, mHealth, review

## Abstract

**Background:**

Headache diaries are often used by headache sufferers to self-monitor headaches. With advances in mobile technology, mobile electronic diary apps are becoming increasingly common.

**Objective:**

This review aims to identify and evaluate all commercially available mobile headache diary apps for the two most popular mobile phone platforms, iOS and Android.

**Methods:**

The authors developed a priori a set of 7 criteria that define an ideal headache diary app intended to help headache sufferers better understand and manage their headaches, while providing relevant data to health professionals. The app criteria were intended as minimum requirements for an acceptable headache diary app that could be prescribed by health care professionals. Each app was evaluated and scored against each criterion.

**Results:**

Of the 38 apps identified, none of the apps met all 7 app criteria. The 3 highest scoring apps, meeting 5 of the app criteria, were iHeadache (developed by Better QOL), ecoHeadache (developed by ecoTouchMedia), and Headache Diary Pro (developed by Froggyware). Only 18% of the apps were created with scientific or clinical headache expertise and none of the apps reported on psychometric properties.

**Conclusions:**

Despite the growing market and demand, there is a concerning lack of scientific expertise and evidence base associated with headache diary apps.

## Introduction

Headache disorders are highly prevalent, with 46% of adults and 51% of children and adolescents presenting with an active headache disorder worldwide [1]. Headache disorders are among the most disabling conditions for both men and women, and a major public health concern [1-3].

Keeping a diary on a regular basis to track headache-related information such as occurrence, symptoms, triggers, and medication intake is often recommended by health care professionals [4,5]. A diary helps both users and health care professionals assess headache impact, make a diagnosis, and inform health care decision making [4,5]. Typically, paper diaries have been used. However, paper diaries can be bulky, data must be entered by hand, and they can be lost or forgotten. Compliance with paper diaries can be a problem, and individuals may be completing multiple diary entries concurrently at a later date, leading to reliability concerns [6]. The limitations of paper diaries, along with recent advances in mobile technology, have led to the increasing adoption of electronic diaries (e-diaries) on mobile devices such as mobile phones [7,8].

The use of mobile e-diaries has several advantages over paper diaries. Mobile e-diaries allow users to conveniently take the diary with them at all times, they make it possible to incorporate branching questions which makes data entry more efficient, and they have the capability of automatically building reports from the data entered, which may help users to identify patterns and predict trends. E-diaries are also beneficial to health care professionals by allowing them to access patient data in real time, verify actual entry times, and ultimately user compliance rates. E-diaries have been shown to be more reliable than paper-based diaries, and they are associated with increased levels of compliance and satisfaction when compared to paper diaries in both adults and children [9,10]. For instance, Stone et al found that the compliance rate for an electronic pain diary was 94%, compared to 11% for a paper diary [6]. They also found that out of the 710 days analyzed, the paper diary was not used on 230 (32%) of the days, yet participants reported a level of compliance over 90% on those days.

E-diaries and other medical apps on mobile phones are rapidly expanding, especially outside the academic setting. The number of available mobile health apps across major mobile phone app stores increased from 17,000 in 2010 to 97,000 apps in 2013 [11,12]. In 2014, 4.55 billion people are expected to use mobile phones overall, with worldwide mobile phone usage predicted to increase by 25% to 1.76 billion people [13]. By 2017, nearly 50% of mobile phones are expected to be smartphones [14]. In addition, it is estimated that by 2015, 500 million mobile phone users will be using a medical app [11].

Concerns are commonly raised around the quality of such mobile health apps, due to the low levels of involvement by health care professionals, and failure to use a scientific evidence base in app development [15,16]. However, no systematic review of many of the available medical apps, including headache diary apps, has been conducted. Recently, Stinson et al systematically reviewed headache diaries used in the research setting only [8]. A previous review by Rosser and Eccleston demonstrated the popularity of pain apps in the commercial app market [16]. They found headache pain was the most common type of pain targeted and found that diary tracking features were included in 24% of the identified pain apps. However, the pain apps were not downloaded and evaluated as part of the review. Since Rosser and Eccleston’s review, the number of headache diary apps has increased dramatically. Our goal was to systematically identify and evaluate all commercially available headache diaries for Apple (iOS) and Android devices. Together, these two platforms represent the majority of devices, with more than 90% (81% Android, 13% iOS) of the global mobile phone market in 2013 [17,18]. The results of this review will help inform health care professionals and potential users on the best available e-diary apps for headache. It will also provide researchers with new electronic assessment tools if apps are found with evidence of reliability and validity. A lack of high-quality apps would demonstrate a need for researchers and health care professionals to improve the existing apps, or develop quality diary apps to fill the current gap in demand.

## Methods

### Search Strategy

The two most popular mobile phone platform app stores were used to identify all available headache diary apps. The Canadian Google Play (Android) and Apple iTunes App (iOS) stores were searched using the following search terms: headache, headache diary, headache tracker, migraine, migraine diary, and migraine tracker. The final app search was conducted on November 2, 2013 by 2 reviewers (ASH, Hayley Stinson, BA). ASH identified a total of 41 apps, while HS identified 42. Agreement between the reviewers was 96.4%. Any discrepancies were resolved by a discussion with a third reviewer (AH).

### Inclusion and Exclusion Criteria

All of the apps identifying themselves in the Canadian Google Play or Apple iTunes App store description as headache logging or tracking tools were included. The apps were then downloaded and excluded from the review if they failed to log or track headaches, despite their associated description. When both a version requiring payment and a free version of an app was available, the version requiring payment was purchased and used, while the free version was excluded. This was done to ensure that the best available version of the app was considered. The apps not available in English were also excluded. Identical apps available in both the Google Play and Apple iTunes App stores were counted only once.

### Data Extraction

One reviewer (ASH) downloaded all of the apps meeting the criteria. The apps were installed on a Google LG Nexus 4 running Android 4.3 and an Apple iPod Touch ME178C/A (4^th^ generation) running iOS 6.1.2. The reviewer extracted the following information for each app: date and version of last update, price, developer, technical requirements, language, assessment schemes (time contingent, signal contingent, or event contingent), presence of reports, reports linking multiple variables, type of reports (plain text, table, graphs/charts), presence of headache entry log (list of previous headache entries), ability to edit previous headache entries, ability to export data from app (eg, email, PDF), reminders, headache characteristics and related variables measured (eg, headache severity, triggers, headache quality), inclusion of customization and personalization features, ability to use the app without Internet connection, the need to create an account to use the app, and presence of advertisements in the app. Any associated components not directly part of the app, such as website components, were not evaluated, given that our main focus was to evaluate the diaries as stand-alone apps.

### App Quality Assessment

#### Overview

Given that no standards exist for evaluating these apps, the authors consensually defined a set of criteria for an ideal headache diary app intended to help headache sufferers better understand and manage their headaches, while providing relevant data to health professionals. Based on the authors’ judgment, an ideal headache diary app should (1) be created with clinical and/or scientific headache expertise, (2) have undergone testing to ensure the diary is a feasible and reliable method of data collection, (3) measure clinically relevant headache variables, (4) be usable, (5) include customizable answer options and reports, (6) include reports linking multiple variables, and (7) have the ability to export headache data from the app. See below for how each of these criteria, intended as minimum requirements for an acceptable headache diary app, were evaluated.

#### App Criterion #1: Apps Created With Headache Expertise

An appropriate app does not necessarily need to be developed by headache experts themselves, but it is important that experts be involved at least in advising development. For this reason, we *a priori* defined that an ideal app be created with headache expertise. The app description available in the app store and any websites linked to the developer, creator, or institution affiliated with app development were examined for scientific or clinical headache expertise. The apps found to be supported by academic or clinical institutions, or created by individuals with MDs or PhDs practicing or doing research in the fields of neurology or pain were considered to have been created with headache expertise. The method used to identify expert involvement was chosen as a feasible strategy. It is possible that headache experts may have been involved in development but not identified in the app descriptions or associated websites. We also acknowledge that headache sufferers can be considered experts in creating diary apps. However, they were not included in this criterion as it was not possible to reliably confirm whether the app creators held this type of expertise.

#### App Criterion #2: Formal Psychometric and Feasibility Testing

To examine whether the feasibility—described in terms of adherence, acceptability, learnability, efficiency, or accuracy—and psychometric properties of the existing apps could have been formally tested, a search of the following databases was conducted: PubMed, Web of Knowledge, and PsychINFO (2000 to October 24, 2013). The search did not include publications prior to 2000; the oldest app versions included in this review were released in 2010. The search terms included “headache or migraine or cephalalgia” and “diary or diaries”. A total of 1442 abstracts were retrieved from our search strategy. Two reviewers (ASH, AH) independently screened all retrieved abstracts (n=723 after removing duplicates) for e-diaries or mobile phone diaries matching the names of the apps, their developers’ names, or descriptions of the content of the apps included in this review. The systematic review of the headache e-dairies developed and used in the academic setting was conducted recently by several members of this research team and was taken into account [8]. Using Cohen’s kappa, the level of agreement between the 2 reviewers screening the abstracts was 1.00, indicating perfect agreement [19,20]. We also acknowledge that the apps may have undergone psychometric or feasibility testing that was not published in the scientific literature. However, it was not possible to verify whether such testing had occurred.

#### App Criterion #3: Clinically Relevant Headache Variables Measured

There is no consensus on a standard set of core variables that should be assessed in a headache diary. Consequently, the authors created and conducted an online survey among headache experts to define what the most clinically relevant headache variables for a headache diary app should be. Headache experts were required to (1) have an MD or PhD, (2) be affiliated with recognized universities, (3) be currently conducting research and/or practicing in the field of neurology or pain, and (4) be published in peer-reviewed journals on the topic of headaches. We identified and invited 35 headache experts to participate. Of the 35 experts contacted, 10 responded. Experts were independently asked to create a list including all variables they believed should be measured in a mobile headache diary. Responses were compiled and comparable responses grouped under the same variable (eg, headache severity, headache intensity, and pain level were grouped together). For a complete list of headache variables recommended by the experts, see Table 1. Those variables suggested by 50% or more of the headache experts were considered clinically relevant. A reviewer (ASH) assessed each of the apps for inclusion of the clinically relevant variables suggested by the experts.

**Table 1:** Headache variables recommended by headache experts (n=10).

Headache variable^a^	Number of experts recommending, n (%)
Headache severity/intensity	10 (100)
Headache triggers	9 (90)
Medication/treatment taken for headache	9 (90)
Associated headache symptoms	7 (70)
Headache frequency (derived from headache occurrence)	6 (60)
Headache-related disability	5 (50)
Headache duration	5 (50)
Response to medication/treatment	3 (30)
Ongoing preventative medication	2 (20)
Time of headache onset	2 (20)
Date of headache	2 (20)
Presence of aura	2 (20)
Menses	2 (20)
Headache pain location	2 (20)
Headache pain quality	2 (20)
Side effects of treatment	1 (10)
Time of treatment	1 (10)
Nonpharmacological treatments	1 (10)
Life events (eg, travel, exercise)	1 (10)
Prodrome symptoms	1 (10)
Sought care from health professionals	1 (10)
Worry/anxiety/fear rating	1 (10)
Stress/mood rating	1 (10)
Sleep rating	1 (10)

^a^Variables recommended by 50% or more of the experts were considered clinically relevant.

#### App Criterion #4: Usable Apps

An ideal app was expected to be usable. Usability is a qualitative attribute which assesses how easy user interfaces are to use and understand [21]. Usability was assessed using a heuristic evaluation, which consists of a small number of expert evaluators assessing the user interface against a list of heuristics, defined as general principles for interaction design [21]. Heuristic evaluation is one of the most common methods of usability assessment. It benefits from being an efficient evaluation method for obtaining high-quality results in a short amount of time, and at a low cost [21-23]. Usability can also be assessed using a variety of methods by users themselves, such as the think aloud protocol, which consists of verbal reports from users [23].

In the current review, each app user interface was systematically inspected, and its compliance with a common list of 10 well-established usability heuristics (see Table 2 for a description of each heuristic) was judged by trained reviewers [21]. Each app user interface was rated on a scale of 1 to 5 (1=poor, 5=excellent) against each of the 10 heuristics and a total usability score was obtained by summing the individual scores (maximum score of 50). The calculated usability score for each app was not intended to be used as a precise indicator of its usability; instead it was intended to be used as an approximate indicator, with higher scores indicating higher perceived usability. One reviewer (ASH) was trained for usability evaluation and evaluated all included apps. For the purpose of exploring interrater reliability, a second reviewer (MW), a software developer with expertise in developing medical apps and testing usability, evaluated the usability of a random selection (9/38, 24%) of the apps. Usability scores are subjective and slight variation between reviewers is expected. Interrater reliability of the total usability scores was assessed using a two-way mixed, absolute agreement, average-measures intraclass correlation (ICC) [24]. Unlike kappa, ICC incorporates magnitudes of disagreement, making it more suitable for evaluating interrater reliability of ratio variables [24]. The resulting ICC was .95, indicating excellent agreement between reviewers [25]. Given that strong agreement was identified between reviewers, it was not considered necessary for the second reviewer (MW) to evaluate more than 24% of the apps. For all of the apps, the first reviewer’s (ASH) scores were used in the presented data. Usability scores of 75% (equivalent to a score of 37.5 out of a maximum score of 50) or higher were considered acceptable for meeting the app criteria.

**Table 2:** Nielson usability heuristics [21].

Heuristic	Description
Visibility of system status	The system should always keep users informed about what is going on, through appropriate feedback within reasonable time.
Match between system and the real world	The system should speak the user’s language, with words, phrases and concepts familiar to the user, rather than system-oriented terms. The system should follow real-world conventions, making information appear in a natural and logical order.
User control and freedom	Users often choose system functions by mistake and will need a clearly marked "emergency exit" to leave the unwanted state without having to go through an extended dialogue. The system should support undo and redo.
Consistency and standards	Users should not have to wonder whether different words, situations, or actions mean the same thing. The system should follow platform conventions.
Error prevention	Even better than good error messages is a careful design which prevents a problem from occurring in the first place. Either eliminate error-prone conditions or check for them and present users with a confirmation option before they commit to the action.
Recognition rather than recall	Minimize the user's memory load by making objects, actions, and options visible. The user should not have to remember information from one part of the dialogue to another. Instructions for use of the system should be visible or easily retrievable whenever appropriate.
Flexibility and efficiency of use	Accelerators—unseen by the novice user—may often speed up the interaction for the expert user such that the system can cater to both inexperienced and experienced users. The system should allow users to tailor frequent actions.
Aesthetic and minimalist design	Dialogues should not contain information which is irrelevant or rarely needed. Every extra unit of information in a dialogue competes with the relevant units of information and diminishes their relative visibility.
Help users recognize, diagnose, and recover from errors	Error messages should be expressed in plain language (no codes), precisely indicate the problem, and constructively suggest a solution.
Help and documentation	Even though it is better if the system can be used without documentation, it may be necessary to provide help and documentation. Any such information should be easy to search, be focused on the user's task, list concrete steps to be carried out, and not be too large.

#### App Criterion #5: Customizable Answer Options and Reports

Customizable answer options are important in making the apps relevant to each user. This feature allows users to create their own inputs when filling out a diary entry. For example, the possibility for the user to add a custom trigger (eg, chocolate, caffeine, or stress) in case the desired trigger does not appear in the default list. To meet this criterion, the apps were required to have at least one headache variable answer option input be customizable and to contain some level of customization in the reports. Customizable reports allow users to better understand their headaches by allowing them to examine the trends that are a concern to them. Examples of customizable reports include controlling the time span of a report or choosing the variables contained in a report. One reviewer (ASH) extracted the required information by reviewing the content of the apps.

#### App Criterion #6: Reports Linking Multiple Variables

Reports allow users to understand trends associated with their headaches. This criterion required that the apps include reports simultaneously linking multiple variables in tables or graphs. For example, a report displaying information about both time of day and headache occurrence was considered to be a report linking multiple variables (time of day and headache occurrence). One reviewer (ASH) extracted the required information by reviewing the content of the apps.

#### App Criterion #7: Ability to Export Headache Data From App

The final app criterion required that the apps include an export feature, allowing users to export logged headache data directly to email, PDF, etc, and allowing the data to be viewed and saved outside the app. This feature is important as it facilitates sharing users’ headache data with their health care professionals. One reviewer (ASH) extracted the required information by reviewing the content of the apps.

## Results

### Overview

In total, 38 apps were identified as headache diaries. For a list of included apps and their characteristics see Table 3. Of the 38 apps, 24 (63%) were available on iOS only, 11 (29%) were available on Android only, and 3 (8%) were available across both platforms. Of the apps identified, 19 (50%) were free, while 19 (50%) required purchase. The average price among the paid apps was Can $2.74. All of the apps used an event-contingent assessment scheme and focused only on tracking headache episodes; none gathered data on days when no headache events occurred. Only 2 apps (5%) included the ability to set reminders.

**Table 3:** Available headache diary apps (n=38) and their characteristics, ordered by number of app criteria met.

Name	Platform/ Version tested	Price, Can$	App criteria							Number of app criteria met (out of 7)
			Created with headache expertise	Published in scientific literature	Headache variables measured / Clinically relevant variables measured (out of 7)	Usability score (%)	Custom answer options^a^ / Custom reports	Reports^b^ / Reports linking multiple variables	Export data from app	
iHeadache	iOS/1.45	4.99	Yes	No	8/7	90	Few/Yes	Yes/No	Yes	5
Headache Diary (ecoHeadache)	iOS/2.3	1.99	No	No	13/7	94	Many/Yes	Yes/Yes	Yes	5
Headache Diary Pro^c^	Android/3.7	2.99	No	No	10/7	82	Few/Yes	Yes/Yes	Yes	5
Headache Diary Pro^d^	iOS/1.5	3.99	No	No	12/7	94	Many/No	Yes/Yes	Yes	4
Migraine Diary	iOS/2.4.1	1.99	No	No	10/7	90	Many/No	Yes/Yes	Yes	4
PainCal	iOS/2.0	1.99	No	No	9/6	80	Many/Yes	Yes/Yes	Yes	4
A Migraine Diary for You	iOS&Android /1.1.3 & 1.1	4.99 / 4.95	No	No	13/7	62	Many/Yes	Yes/Yes	Yes	4
Migraine	iOS/1.1	Free	Yes	No	7/6	72	Few/No	Yes/Yes	Yes	3
Migralex	iOS/1.1	Free	Yes	No	10/5	84	Few/No	Yes/No	Yes	3
Oh, My head	iOS/1.0	1.99	No	No	5/2	76	Few/Yes	Yes/Yes	No	3
Migraine Free	iOS/1.1	Free	Yes	No	5/5	74	None/No	Yes/Yes	Yes	3
Cluster Headaches	Android /2.0.04	Free	No	No	4/3	80	Few/No	Yes/Yes	Yes	3
Headache Relief Log	Android/1.07	1.00	No	No	7/4	80	Few/No	Yes/Yes	Yes	3
Headache App	iOS&Android /1.4.5 & 1.5	Free	No	No	9/6	76	Many/No	Yes/Yes	Yes	3
Headache Note^e^	iOS&Android /1.2.1 & 1.0.3	Free	No	No	6/5	84	Few/No	Yes/Yes	Yes	3
Migraine Meter	iOS/2.6	Free	Yes	No	9/7	82	Few/No	Yes/No	Yes	3
PainTrek	iOS/1.1	Free	Yes	No	8/5	66	None/Yes	Yes/Yes	No	2
American Migraine Foundation	iOS/1.0.1	Free	Yes	No	7/5	74	No/No	No/No	Yes	2
PainCalendar	iOS/1.0	5.99	No	No	9/6	86	Few/No	Yes/Yes	No	2
Headache & Migraine Diary	iOS/2.5.0	1.99	No	No	5/3	74	No/Yes	Yes/Yes	Yes	2
Migraine Journal	iOS/0.9.1	Free	No	No	4/4	86	Few/No	No/No	Yes	2
MigraineMate	iOS/2.198	Free	No	No	3/2	94	No/No	Yes/Yes	No	2
Migraine Diary	iOS/1.0	Free	No	No	8/6	86	No/No	No/No	Yes	2
Headache^f^	iOS/1.1.0	4.99	No	No	6/4	82	Few/No	Yes/Yes	No	2
Migraine Stop	iOS/1.0	Free	No	No	9/6	92	No/No	Yes/No	Yes	2
Migraine tracker!	iOS/4.0	Free	No	No	2/1	82	No/No	No/No	No	2
Headache diary^g^	Android/1.3.0	1.34	No	No	7/6	72	Few/No	Yes/Yes	Yes	2
Migraine Calendar	Android/4.0	1.96	No	No	10/6	86	Few/No	Yes/No	Yes	2
HeadacheDiary	Android/1.0	2.04	No	No	6/6	76	No/Yes	Yes/Yes	No	2
Headache Diary^h^	Android/1.25	Free	No	No	7/4	74	Few/No	Yes/Yes	Yes	2
MyGraine	iOS/1.1	Free	No	No	9/5	70	Few/No	Yes/Yes	Yes	2
Migraine Institute	iOS/1.4	0.99	Yes	No	8/6	74	No/No	No/No	Yes	2
Ubiqi Health Migraine Tracker	Android/2.1	Free	No	No	7/5	82	Many/No	No/No	No^i^	1
Migraine Tracker	Android/0.9b	Free	No	No	4/3	86	No/No	No/No	No	1
My Headache Diary	iOS/1.1	Free	No	No	7/5	72	No/No	No/No	No	0
migraineDiary	Android/0.1	Free	No	No	5/4	72	No/No	No/No	No	0
Headache Diary^j^	Android/1.1	0.99	No	No	6/5	64	No/No	No/No	No	0
Headache & Migraine Tracker Pro	iOS/1.2	0.99	No	No	5/3	0^k^	Few/No	No/No	No	0

^a^Few: 3 or less headache variables allow for custom answer options; Many: more than 3 headache variables allow for custom answer options.

^b^Refers to the app’s ability to create reports in general, not necessarily custom reports or reports linking multiple variables.

^c^Developed by Froggyware.

^d^Developed by appcellent GmbH.

^e^Full name: Headache Note – You can manage headache by recording the pain and the taken medicine (iOS name); Headache Note-be healthier- (Android name).

^f^Full name: Headache – migraine and headache journal/log/calendar.

^g^Developed by Marcel Shroder.

^h^Developed by Benjamin Gerfelder.

^i^Able to export data from website component associated with app.

^j^Developed by Tmoney.

^k^Not usable; unable to load headache entries.

### App Quality: App Criteria

#### Overview

**Figure 1:**
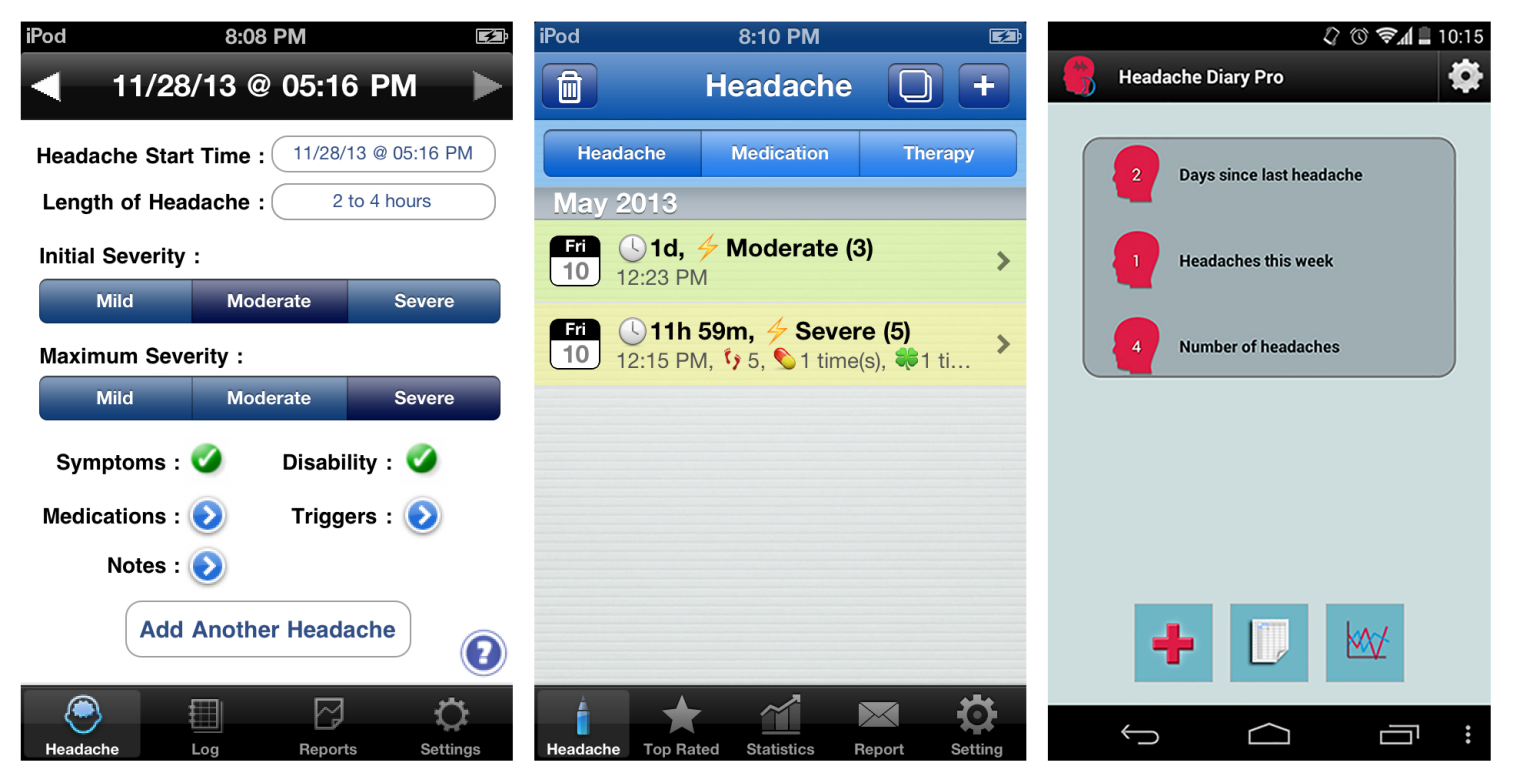
The home screen of iHeadache (left), ecoHeadache (middle), and Headache Diary Pro (right).

The quality of the apps was determined by how many app criteria were met. The apps with the highest quality were iHeadache (developed by Better QOL), ecoHeadache (developed by ecoTouchMedia), and Headache Diary Pro (developed by Froggyware), each of which met 5 of the 7 app criteria. See Figure 1 for a screenshot of the 3 highest scoring apps. Only 7 of the 38 available apps met 4 or more of the app criteria. The median number of app criteria met was 2. Table 4 shows the number of apps meeting each criterion.

**Table 4:** Number of apps meeting each app criterion (N=38).

Criterion	Number of apps meeting the criterion, n (%)
1. Created with headache expertise	7 (18)
2. Formal psychometric and feasibility testing	0 (0)
3. Clinically relevant headache variables measured	7 (18)
4. Usable	24 (63)
5. Customizable answer options and reports	9 (24)
6. Reports linking multiple variables	22 (58)
7. Export headache data from app	25 (66)

#### App Criteria #1 and #2: App Created With Headache Expertise and Formal Feasibility and Psychometric Properties Testing

Only 7 apps (18%) were found to have been created with scientific or clinical expertise and met criterion #1. None of the apps in this review were found in the scientific literature search, and as a result none of the apps were considered to have undergone formal psychometric or feasibility testing (criterion #2).

#### App Criterion #3: Clinically Relevant Headache Variables Measured

Of the 38 apps, 7 of them (18%) measured all 7 clinically relevant headache variables as defined by app criterion #3. The average number of headache variables measured in each app was 7 out of 24. The average number of variables measured per app that were identified as clinically relevant by the experts was 5 out of 7. The most common variable measured was headache intensity (37/38, 97%), followed by medication usage (30/38, 79%), triggers (27/38, 71%), time of headache (27/38, 71%), notes/comments (26/38, 68%), other headache symptoms (25/38, 66%), headache duration (25/38, 66%), location of headache (21/38, 55%), headache disability (12/38, 32%), headache quality (11/38, 29%), and other coping strategies (11/38, 29%). Other variables less frequently measured were geographical location, weather, mood, and headache type. For a complete list of headache variables measured by those apps meeting 4 or more app criteria, see Table 5.

**Table 5:** Headache variables measured by all apps (n=7) meeting 4 or more app criteria.

Headache variables measured	App						
	iHeadache	Headache Diary (ecoHeadache)	Headache Diary Pro^a^	Headache Diary Pro^b^	Migraine Diary	PainCal	A Migraine Diary for You
Headache severity/intensity	✓	✓	✓	✓	✓	✓	✓
Headache triggers	✓	✓	✓	✓	✓	✓	✓
Medication/treatment taken for headache	✓	✓	✓	✓	✓	✓	✓
Associated headache symptoms	✓	✓	✓	✓	✓	✓	✓
Headache frequency	✓	✓	✓	✓	✓	✓	✓
Headache-related disability	✓	✓	✓	✓	✓		✓
Headache duration	✓	✓	✓	✓	✓	✓	✓
Time of headache onset	✓	✓	✓	✓	✓	✓	✓
Headache pain location		✓	✓	✓	✓	✓	✓
Headache pain quality		✓		✓		✓	✓
Nonpharmacological treatment and coping strategies		✓		✓	✓		✓
Type of day (eg, work, school)		✓					
Type of headache		✓	✓				
Weather when headache occurred							✓
Activity when headache occurred							✓
Geographical location when headache occurred				✓			

^a^Developed by Froggyware.

^b^Developed by appcellent GmbH.

#### App Criterion #4: Usable Apps

Of the 38 apps, 24 (63%) met this criterion, which consisted of scoring a total usability score of at least 75%. Usability scores ranged from 0% to 94% with a median score of 80%.

#### App Criteria #5, #6, and #7: Customizable Answer Options and Reports, Reports Linking Multiple Variables, and Ability to Export Headache Data From App

Most of the apps (27/38, 71%) contained reports on headache data, with 58% (22/38) of the apps containing reports linking multiple variables, while customizable reports were less common (9/38, 24%). The ability to modify existing lists of answer options for a headache variable (eg, adding a new trigger to the preexisting list) was seen in 63% (24/38) of the apps. Many of the apps (25/38, 66%) also allowed data entered into the diary to be exported, often via email or by creating PDF documents.

## Discussion

### Available Apps

Clinicians often recommend that headache sufferers use a diary to record headache events, and e-diaries have been growing in popularity. Despite this, e-diaries created and tested by headache experts in academic settings are not available to the general population. As a result, consumers are restricted to what is available in the app stores. Despite the large volume of apps available commercially, none of the apps met all 7 app criteria. It is especially concerning that none of the apps identified in this review were found to have undergone formal feasibility or psychometric property testing. It is essential when developing mobile health apps to test feasibility and, later on, psychometric properties in order to offer consumers high-quality assessment tools. Additionally, only 2 apps included the ability to set reminders, despite research demonstrating that reminders can increase adherence in health interventions [26,27]. Overall, this review has demonstrated the lack of quality headache diary apps available to consumers.

Of the 3 highest scoring apps (iHeadache, ecoHeadache, and Headache Diary Pro), iHeadache, developed by Better QOL for iOS, was the only app created with scientific or clinical headache expertise and is available for Can $4.99. The app records all clinically relevant variables without recording other nonessential information, making it easy to use with fast data input. However, it has not been formally tested for feasibility or psychometric properties and the in-app reports are in plain text format that can be difficult to interpret. The app developed by ecoTouchMedia for iOS, ecoHeadache, is available for Can $1.99. While it offers good levels of customization, it tracks significantly more information than what has been defined as essential. This app can track 13 headache variables and can generate 24 chart reports, in addition to customizable reports. Headache Diary Pro, developed for Android by Froggyware, costs Can $2.99 but was not rated as usable as were the 2 other apps mentioned above. However, it was the highest rated Android offering.

### Recommendations and Future Directions

A long-term strategy is needed to begin offering validated evidence-based medical apps to the general population. As a first step it is essential to disseminate the state of the current apps to headache sufferers and their health care professionals. Currently, this can be done through educating health care professionals on the existing app environment, allowing them to inform patients. In addition, findings can be distributed using social media to educate consumers on the quality of existing apps. Given the fast-growing number of medical apps available, it is not realistic to propose regulating the full marketplace. As well, systematic reviews such as this will become more complex as the number of apps increases, especially taking into account the rate at which apps are being developed and upgraded.

We recommend that headache experts and the research community partner with app developers to test high-quality, popular apps currently available to consumers. Another solution would be giving developers the opportunity to have their apps evaluated by an independent third party organization with mobile health expertise. There are current initiatives moving in this direction, for example, the National Health Service (NHS) in the United Kingdom has begun reviewing medical apps and currently offers a growing list of approved apps online [28]. In addition, the United States Food and Drug Administration (FDA) recently released its recommendations for medical apps [29]. The FDA will regulate only those apps that can be used as an accessory to regulate a medical device (eg, an app that controls the delivery of insulin through a pump), or those apps that are similar to currently regulated medical devices, by transforming a mobile platform into a medical device using attachments (eg, attachment of electrocardiograph electrodes to a mobile platform).

We have evaluated the apps taking into account current knowledge. However, it is critical for apps to advance along with research, which will require continual updates to the apps to satisfy the newest developments and discoveries.

We intend to work toward filling the gaps identified in this review. We are currently developing the Wireless Headache Intervention (WHI) diary app called myWHI. The myWHI diary is designed to meet all 7 app criteria. It has been developed using a participatory design process involving both headache sufferers and headache experts [30]. The app has been shown to be usable and feasible and we are currently testing its psychometric properties [31]. The myWHI diary has been designed to be used as a stand-alone app and will also be offered as part of an online comprehensive cognitive behavioral therapy (CBT) intervention for chronic headaches.

### Strengths and Limitations

Information on pain apps (including headache diaries) has been synthesized in a previous review by Rosser and Eccleston [16]. The app evaluation in Rosser and Eccleston’s review, along with other app reviews [32,33], was limited to the app descriptions, without downloading the apps. The authors of the current review found that the app description can insufficiently, and sometimes incorrectly, describe the app function. In this review, the authors downloaded and used all of the existing headache diary apps for a more comprehensive evaluation.

The scope of the review was limited to the English-language apps available in the Canadian app stores, and looked only at the 2 most popular platforms. Different apps may be available in other countries, and other apps may exist on less popular platforms. This review focused on mobile apps, and did not consider e-diaries available only as general websites. The authors focused on mobile diary apps because they are portable, which is key for a self-monitoring tool, allowing users to use them on the go. This in turn may facilitate increased adherence [34,35]. However, the development and sustainability of mobile apps may be more economically expensive, especially when apps must be developed for multiple platforms [36].

The app evaluation method had several limitations. First, the method for evaluating the quality of these apps was developed by the authors and the criteria have not been validated. Second, the app criteria were each given equal weight in evaluating the apps, despite the fact that some criteria might be more significant than others in terms of the effectiveness of the app. Third, we made the assumption that the more app criteria met, the higher the app quality, but this may not be the case. Fourth, when reaching a consensus on the most relevant clinical variables that should be collected by a headache diary, only scientific or clinical experts were used—headache sufferers were not consulted and may have suggested other relevant variables. Fifth, in determining if the app was created with headache expertise the authors were limited to the information made available to them in the app store description and developer websites, and these descriptions can be of poor quality. Finally, the literature search seeking to identify formal feasibility or psychometric testing of the apps could not confirm that this type of testing had not occurred, only that it has not been published.

The limitations to this review reflect limitations and concerns with the medical app market in general. It is an emerging field lacking quality standards with poor transparency in the app development process.

### Conclusions

In summary, although a proliferation of headache diary apps exists, the majority do not meet reasonable quality standards. More emphasis on the quality of these tools is needed as they are easily accessed and used by the general population, often for self-managing health conditions. The demand remains for a high-quality, evidence-based headache diary app.

## Abbreviations

CBT: cognitive behavioral therapy
FDA: Food and Drug Administration
ICC: intraclass correlation
WHI: Wireless Headache Intervention
